# Machine learning-derived identification of tumor-infiltrating immune cell-related signature for improving prognosis and immunotherapy responses in patients with skin cutaneous melanoma

**DOI:** 10.1186/s12935-023-03048-9

**Published:** 2023-09-26

**Authors:** Shaolong Leng, Gang Nie, Changhong Yi, Yunsheng Xu, Lvya Zhang, Linyu Zhu

**Affiliations:** 1 Department of Dermatovenereology, The Seventh Affiliated Hospital of Sun Yat-Sen University, Shenzhen, China; 2 Department of Interventional Radiology, Cancer Hospital of Shantou University Medical College, Shantou, China

**Keywords:** Skin cutaneous melanoma, Machine learning, Tumor microenvironment, Immunotherapy

## Abstract

**Background:**

Immunoblockade therapy based on the PD-1 checkpoint has greatly improved the survival rate of patients with skin cutaneous melanoma (SKCM). However, existing anti-PD-1 therapeutic efficacy prediction markers often exhibit a poor situation of poor reliability in identifying potential beneficiary patients in clinical applications, and an ideal biomarker for precision medicine is urgently needed.

**Methods:**

10 multicenter cohorts including 4 SKCM cohorts and 6 immunotherapy cohorts were selected. Through the analysis of WGCNA, survival analysis, consensus clustering, we screened 36 prognostic genes. Then, ten machine learning algorithms were used to construct a machine learning-derived immune signature (MLDIS). Finally, the independent data sets (GSE22153, GSE54467, GSE59455, and in-house cohort) were used as the verification set, and the ROC index standard was used to evaluate the model.

**Results:**

Based on computing framework, we found that patients with high MLDIS had poor overall survival and has good prediction performance in all cohorts and in-house cohort. It is worth noting that MLDIS performs better in each data set than almost all models which from 51 prognostic signatures for SKCM. Meanwhile, high MLDIS have a positive prognostic impact on patients treated with anti-PD-1 immunotherapy by driving changes in the level of infiltration of immune cells in the tumor microenvironment. Additionally, patients suffering from SKCM with high MLDIS were more sensitive to immunotherapy.

**Conclusions:**

Our study identified that MLDIS could provide new insights into the prognosis of SKCM and predict the immunotherapy response in patients with SKCM.

**Supplementary Information:**

The online version contains supplementary material available at 10.1186/s12935-023-03048-9.

## Introduction

Skin cutaneous melanoma (SKCM) is a highly heterogeneous and highly aggressive malignant tumor, which progresses quickly and has a high fatality rate, which seriously endangers human health. The morbidity of SKCM have increased in the past decades [1, 2]. Fortunately, with the advancement of science and technology, many studies have found that PD-1 (programmed cell death protein 1) immune checkpoint blockade therapy can significantly improve clinical efficacy and patient survival through high-level anti-SKCM response induction [3, 4]. However, studies have also found that only a minority of patients in immunotherapy tend to have a good clinical response to PD-1 blockade therapy, with most patients not achieving significant therapeutic effects and a small proportion even experiencing severe unexplained immune side effects. Moreover, the high cost of immune checkpoint inhibitors undoubtedly further increases the financial burden on patients [5, 6]. Therefore, the means and modalities used to improve the effectiveness of clinical use of anti-PD-1 drugs in the course of immune blockade therapy have become a major clinical issue to avoid ineffective treatment, improve patient survival and reduce the medical burden on patients.

Biomarkers provide an effective means for disease staging, new drug evaluation, and efficacy assessment. Molecular biomarkers such as TMB (tumor mutation burden), CTLA-4 (cytotoxic T lymphocyte-associated antigen-4), PD-1/PDL1 (Programmed cell death 1 ligand 1) have been gradually incorporated into clinical guidelines. However, these markers have some limitations. A large number of patients with positive PD-L1 protein expression (at least 40–50%) did not respond positively and objectively to PD-1 blockade therapy. In contrast, patients with negative PD-L1 protein expression (~ 15%) responded well [7]. Moreover, even though high TMB characteristics were associated with overall survival, they were likely not associated with an objective response to PD-1 blockade therapy [8]. Despite the progress in the study of PD-L1 protein expression and TMB as predictive markers of anti-PD-1 therapy, however, these two markers still have great deviation, even contradictory results, in predicting the degree of benefit of PD-1 blockade and the appropriateness of treatment [9, 10]. Inappropriate molecular biomarkers will delay the optimal time of treatment and lead to heavy social and economic burden. To solve this problem, many multi-gene signatures based on specific pathways have been developed (m6a, miRNA, lncRNA) [11–13]. Although it was validated using a public database, the inadequacy of modeling methods, and the lack of rigorous validation limit their wide application in clinical practice.

The development of new markers with better predictive performance or the establishment of a comprehensive rubric consisting of multiple predictive markers are effective strategies to overcome these problems. However, many related studies tend to focus only on the expression level of single or multiple genes, while neglecting the functional relevance of gene co-expression or gene co-expression processes and the deeper important characteristic information presented by these functional gene networks [14, 15]. Therefore, we attempted to apply 10 machine learning algorithms to construct a machine learning-derived immune signature (MLDIS) in 712 SKCM patients. We further validated the clinical applicability value of our signature as well as its robust performance for predicting prognosis by comparing it with 51 published signatures, traditional clinical traits, and molecular features. The established MLDIS can stratify patients with SKCM and predict the outcome of immunotherapy. In summary, our study offers an important reference for achieving early diagnosis, prognostic evaluation, stratified management, individualized treatment, and improving the clinical outcomes of patients with SKCM.

## Methods

### Data acquisition

The mRNA expression data of SKCM was further retrieved by searching the GEO database with the following keywords: “skin cutaneous melanoma”, “SKCM”, and “melanoma”. To ensure the quality of the collected data, the data set must contain the patient’s prognostic information and have a valid sample size of not less than 50 patients. After initial screening, 3 gene expression omnibus profiles with prognostic information (GSE22153 [16], GSE54467 [17], and GSE59455 [18]) were selected and downloaded. 57 samples in GSE22153, 79 samples in GSE54467, and 141 samples in GSE59455. Subsequently, the gene expression data obtained by screening were cleaned, and the data of multiple probes corresponding to the same gene were averaged and combined. We also adopted TCGA public database (N = 457). For immunotherapy cohorts, we enrolled 6 cohorts treated with immunotherapy: IMvigor cohort [19], GSE35640 [20], GSE91061 [21], GSE78220 [22], Van Allen [23], and Nathanson [24].

### Data preprocessing

The Ensemble ID was converted into gene symbol. Next, A FPKM gene expression matrix was acquired from TCGA and converted into TPM format [25]. The merged expression matrix was then eliminated from batch effects and normalized using the R package “sva” [26].

### Immune cell infiltration analysis

SsGSEA method was used to evaluate the content of 28 immune cells in each tumor tissue sample [27]. To avoid computational errors caused by a single algorithm and different sets of marker genes for tumor microenvironment (TME), we downloaded immune infiltrate data evaluated using the 7 algorithms. Also, we used the ESTIMATE algorithm to calculate the immune score and stromal score.

### Weighted gene co-expression network analysis (WGCNA)

This process uses “WGCNA” packets to identify characteristic genes associated with immune cells. First, a standardized TCGA-SKCM gene expression profiling was prepared, and then the genes were sequenced according to the median absolute deviation (MAD) to obtain a list of top 5000 genes. Then, the 5000 genes were constructed by gene co-expression network to obtain gene pairs. Then, the Pearson correlation coefficients between each pair of genes were calculated and the adjacency matrix was generated. Then, the adjacent matrix is transformed into topological overlap measure (Tom) by tomlikeity function. The average linkage hierarchical clustering based on Tom dissimilarity is used to cluster genes with similar expression patterns into the same module. The characteristic gene is the main component of each gene module, and it is also the most representative expression mode of the module. Then, we calculated the gene significance (GS) and the gene module significance (MS), and extracted the module genes significantly related to the immune cell phenotype for further analysis [28].

### Functional analysis

To understand the potential functions of module genes, we used the “cluterprofile” R package to perform functional analyses of KEGG and GO. Adjusted P values < 0.05 for the GO pathway and KEGG pathway were considered statistically significant [29].

### Identify hub genes

To explore differences in expression levels of module genes between normal and tumor patients, this study used “limma” packages to perform mRNAs differential expression analysis based on datasets TCGA-SKCM and GTEx [30]. Among them, RNAs with adjusted P-value < 0.05 and |log_2_FC|≥ 1 was considered statistically significant differentially expressed module genes. Next, to further verify the expression level of module genes and prognostic value. We used "survival" package to perform univariate Cox analysis and multivariate Cox analysis on the module genes. Among them, RNAs with P-value < 0.05 were considered statistically significant hub genes. To explore the predictive power of hub genes for prognosis, the area under curve (AUC) of each hub genes was calculated based on the expression data of each hub gene using the independent data set TCGA-SKCM using the “pROC” package [31].

### Molecular subtyping of hub genes in a meta-cohort

The meta-cohort samples were classified into different molecular subtypes by consensus clustering based on endoplasmic reticulum stress genes through “Consensus cluster plus” package, use the cumulative distribution function cumulative distribution function to help select multiple clusters and achieve cluster stability [32].

### Machine learning-derived immune signature (MLDIS)

**Fig. 1:**
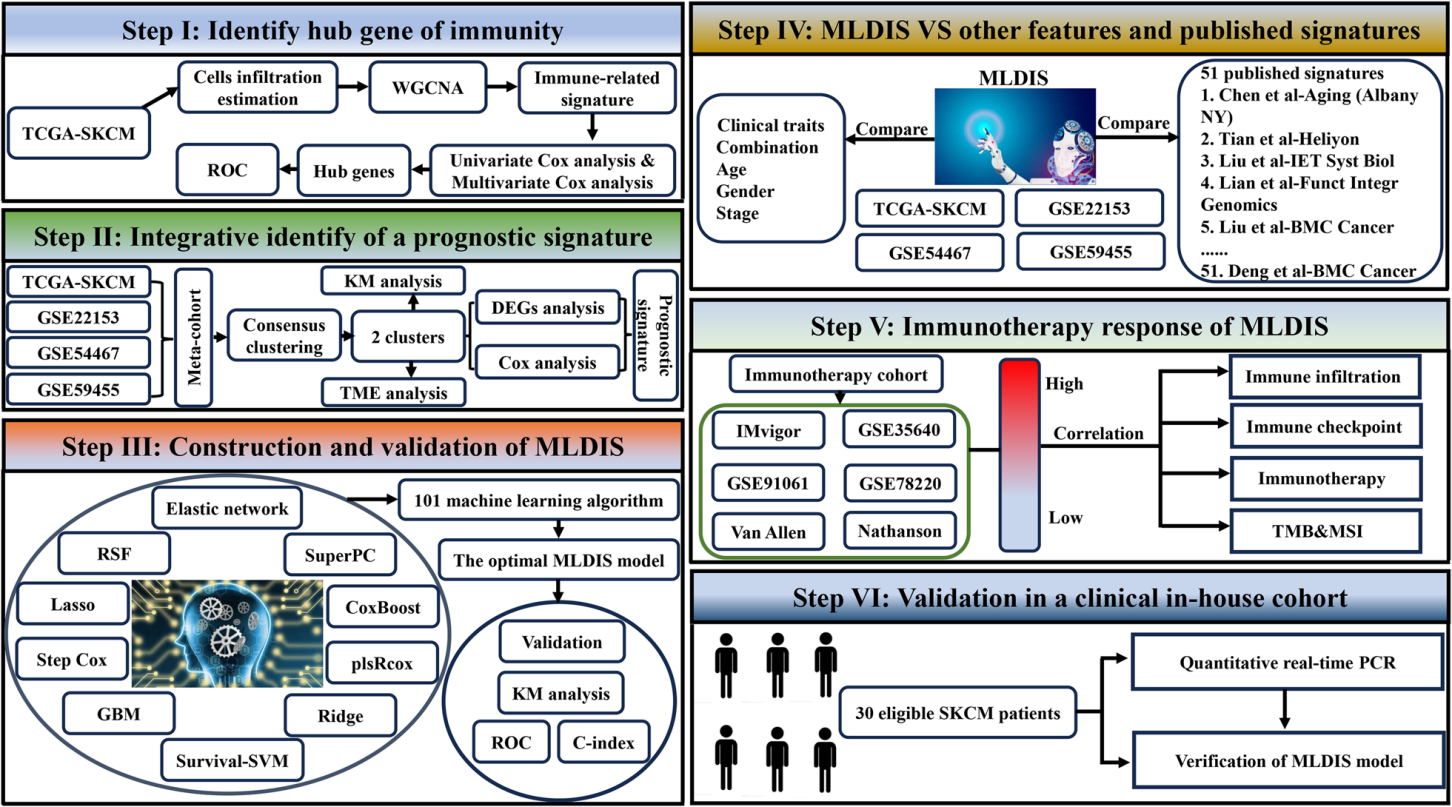
Flow chart of the study

The overall workflow of our study is presented in Fig. 1. To construct a MLDIS model for SKCM, firstly, we identified different expression genes and prognosis genes between 2 subtypes. Then, a prediction model was fitted using 101 algorithm combinations. The initial signature discovery was performed in TCGA-SKCM. All the pairs of are formed and the one with the best C-index value is identified as the optimized parameters. Finally, the independent data set (GSE22153, GSE54467, and GSE59455) is used as the verification set, and the ROC index standard is used to evaluate the model.

### Patients

The human SKCM tissues were from the Seventh Affiliated Hospital of Sun Yat-sen University collected from May 2021 to July 2022. This study was approved by the Ethics Review Board of the Seventh Affiliated Hospital of Sun Yat-sen University. All experiments complied with the relevant regulations, and all patients provided written informed consent. All patients were aged 18 years or older, and received available standard systemic therapies. The clinicopathological data of those patients was in Additional file 1: Table S1.

### qRT-PCR

Total RNA from tissues was isolated using TRIzol (Invitrogen, Canada) reagent, the specific operation is carried out with reference to the instructions for the operation of the kit. RNA (1 μg) was converted into cDNA using the RevertAid First Strand cDNA Synthesis Kit (Takara, China). qRT-PCR was performed using SYBR Green Mixture (Takara, China) in the ABI Step One-Plus System (ABI7500, USA). Target gene expression was normalized against GAPDH. Th primer sequences was in Additional file 2: Table S2.

### Immunohistochemistry (IHC) staining

Tumor tissue was paraffin-embedded and cut into 4-µm cross-sections. After dewaxing, antigen repair and blocking, sections were incubated with anti-CD8 (1:200, CST, Shenzhen), anti-PD-1 (1:200, CST, Shenzhen), and PD-L1 (1:200, CST, Shenzhen) for 2 h at 37 °C. The tissue was then incubated with biotin-labeled goat anti-rabbit secondary antibody for 20 min, followed by incubation with HRP-labeled streptavidin for 10 min at 37 °C. After washing with PBS, the nuclei were stained using hematoxylin solution. We have added it. Thank you.

### Statistical analysis

This study is based on R (4.2.2) software for statistical analysis. Wilcoxon test was used for comparison of two groups, and Kruskal–Wallis’s test was used for comparison of multiple groups. In univariate and multivariate Cox regression analysis of genes, HR (hazard ratio) > 1 represents a risk factor for prognosis and HR < 1 represents a protective factor for prognosis. Correlations between variables were explored using Spearman or Pearson coefficients. We performed Kaplan–meier survival analysis using the R package “Survival”. The significance level was set at P < 0.05, and all statistical tests were two-sided.

## Results

### Screening of key immune cell-related gene modules in TCGA-SKCM

**Fig. 2:**
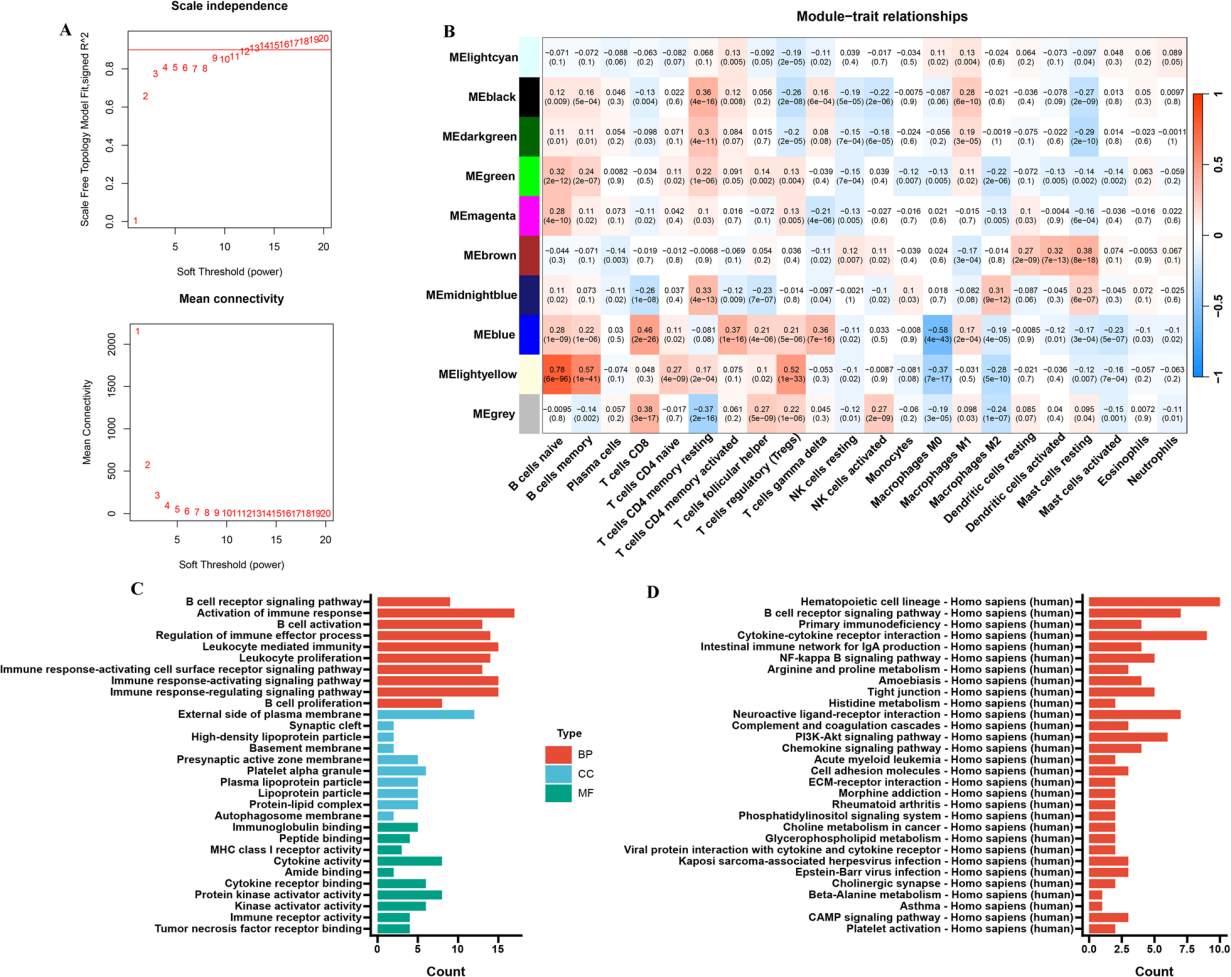
Screening of key immune cell-related gene modules in TCGA-SKCM. **A** Analysis of network topology for different soft-threshold power. The top panel shows the impact of soft-threshold power on the scale-free topology fit index; the bottom panel displays the impact of soft-threshold power on the mean connectivity. **B** Correlation analysis between module eigengenes and immune cells. **C** GO enrichment analysis on the module genes. (D) KEGG enrichment analysis on the module genes

To identify key modules of genes associated with immune cells, we calculated 28 immune cells infiltration assessed by ssGSEA, and then the construction results of WGCNA were obtained. Figure 2A is the result of screening for the soft threshold parameter β, in which the soft threshold parameter β = 12 (scale-free R^2^ = 0.90) was used to ensure that the constructed gene network was scale-free network. The average linkage hierarchy clustering identified 10 gene modules (Additional file 5: Fig. S1A and B). The most relevant to immune cells was the light-yellow gene module (Cor = 0.78, P < 0.001), which contained 132 key genes (Fig. 2B, Additional file 3: Table S3). GO and KEGG functional enrichment analysis was performed on the 132 key module genes mentioned above, the results showed that the functions of these genes were mainly related to immune regulation (such as activation of immune response, regulation of immune effector process and B cell receptor signaling pathway), hematopoiesis, and signal transduction (Fig. 2C and D). Activating immune cells by blocking immune checkpoints and enhancing the anti-tumor response of the immune system are key factors in the therapeutic efficacy of anti-PD-1 immunotherapy, these results suggest that the genes involved in the regulation of immune function may be related to the efficacy of immunotherapy.

### Identify hub genes in TCGA-SKCM

**Fig. 3:**
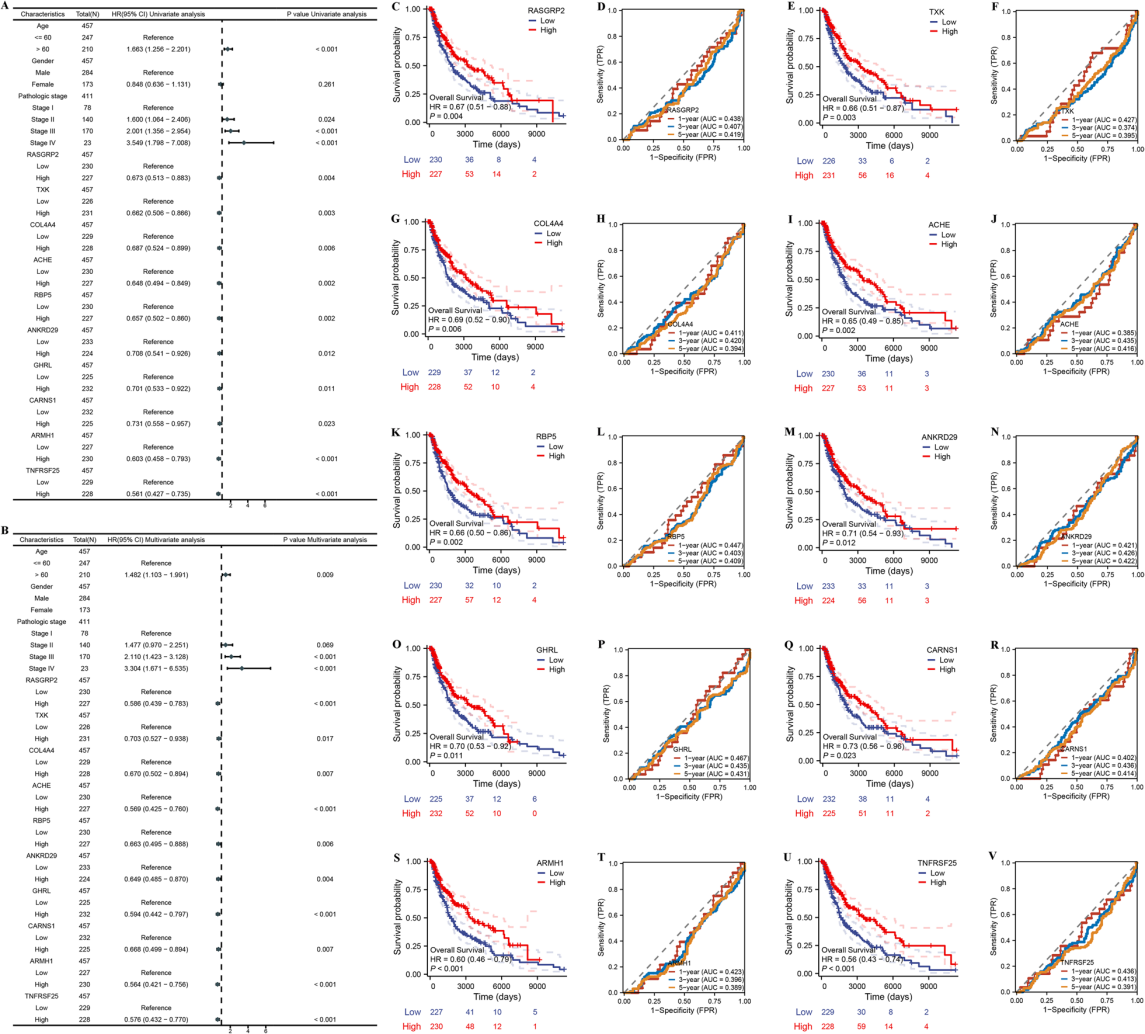
Identify hub genes in TCGA-SKCM. **A** Univariate Cox analysis and **B** multivariate Cox analysis identified 10 prognostic RNAs in the TCGA-SKCM cohort (n = 457). Kaplan–Meier curves of OS for the RASGRP2 (**C**), TXK (**E**), COL4A4 (**G**), ACHE (**I**), RBP5 (**K**), ANKRD29 (**M**), GHRL (**O**), CARNS1 (**Q**), ARMH1 (**S**), TNFRSF25 (**U**) in the TCGA-SKCM cohort (n = 457). Time-dependent ROC analysis for predicting OS at 1, 3, and 5 years for the RASGRP2 (**D**), TXK (**F**), COL4A4 (**H**), ACHE (**J**), RBP5 (**L**), ANKRD29 (**N**), GHRL (**P**), CARNS1 (**R**), ARMH1 (**T**), TNFRSF25 (**V**) in the TCGA-SKCM cohort (n = 457). Data are presented as hazard ratio (HR) ± 95% confidence interval [CI]

Next, in order to identify module genes that are important biological regulators of genes associated with immune cells, we compared the expression of 132 module genes in TCGA-SKCM tissues and GTEx normal tissues. The results showed that 45 genes were significantly different (Additional file 6: Fig. S2). Next, univariate Cox analysis and multivariate Cox analysis of these genes, and identified 10 genes as possible prognostic markers for SKCM (Fig. 3A and B). Moreover, low expression of 10 genes correlated with worse outcome in SKCM. To explore the prognostic ability of the 10 genes screened above, we performed ROC (receiver operating characteristic curve) analysis. The results showed that these genes did not predict prognosis (AUC < 0.5). It suggests that they may not be potential prognostic predictors (Fig. 3C–V). Therefore, there is an urgent need to develop a more effective model to predict the prognostic of SKCM.

### Development consensus clusters based on 10 hub genes in a meta-cohort

**Fig. 4:**
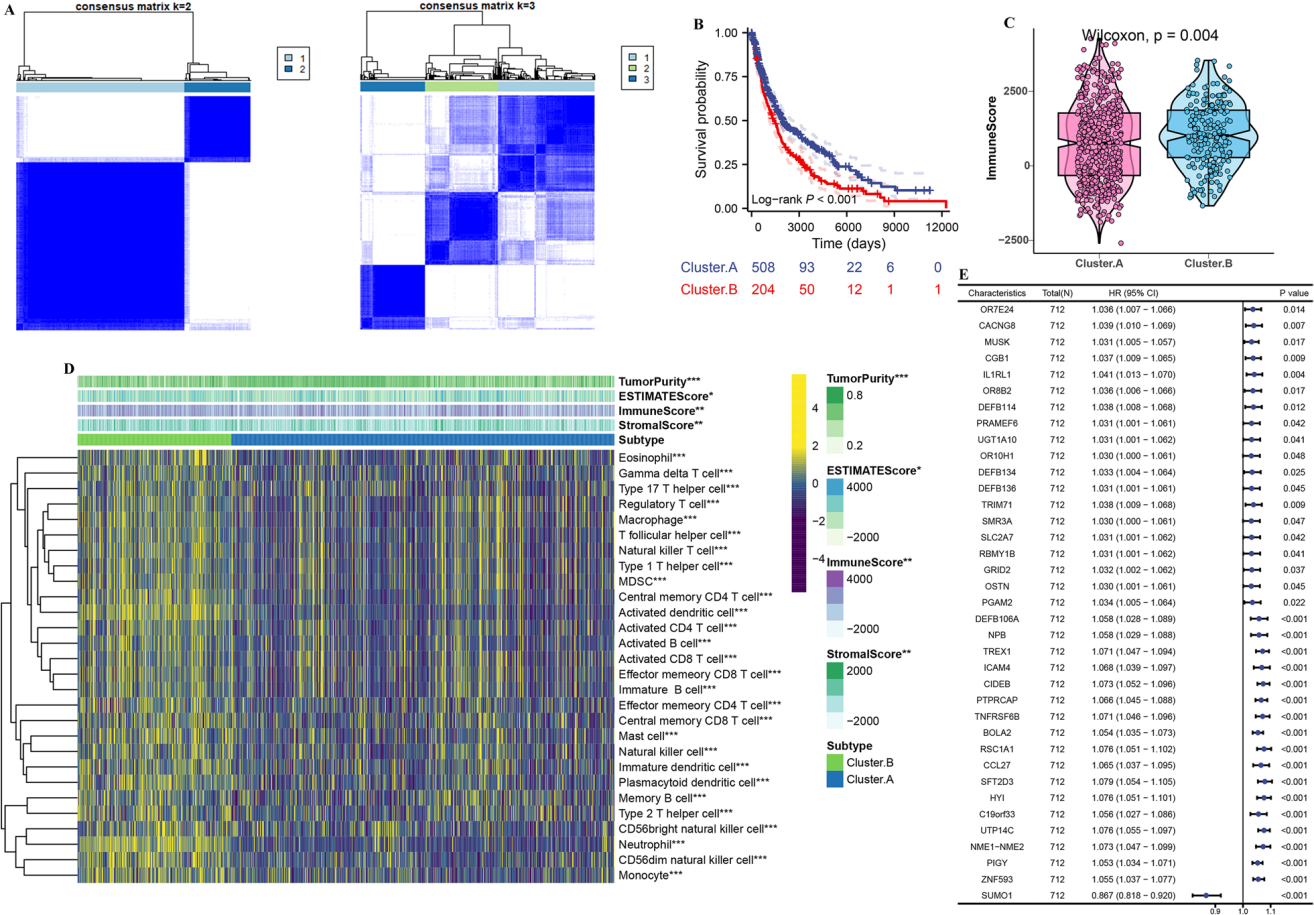
Development consensus clusters based on 10 hub genes in a meta-cohort. **A** The consensus score matrix of all samples when k = 2. A higher consensus score between two samples indicates they are more likely to be grouped into the same cluster in different iterations. **B** Kaplan–Meier curve showed a significant difference between the 2 clusters. **C** The distribution of immune score inferred by ESTIMATE algorithm between 2 clusters in the meta-cohort. **D** The distribution of 28 immune cell subsets infiltration between 2 clusters. **E** Univariate Cox analysis identified 37 prognostic RNAs in the meta-cohort (n = 712). The asterisks represented the statistical p-value (*P < 0.05; **P < 0.01; ***P < 0.001)

First, we merge TCGA-SKCM, GSE22153, GSE54467, and GSE59455 into a meta-cohort. Then, all tumor samples were divided into k (k = 2 to 9) different subtypes. The results of cluster analysis showed that k = 2 was the best cluster (Fig. 4A). Kaplan–Meier analysis showed that cluster.A had better survival outcomes than cluster.B (Fig. 4B). Next, the immune cell infiltration, stromal score, immune score and ESTIMATE score were lower in cluster.A group, but the tumor purity was higher in cluster.A group (Fig. 4C and D). To explore the potential biological change between distinct cluster, we explored its expression level and prognostic value in cluster.A group and cluster.B group. The results showed that the expression of 57 genes were up-regulated in cluster.B group compared to cluster.A group (Additional file 4: Table S4). In addition, survival analysis showed that 36/57 genes, except *SUMO1*, were significantly associated with poor outcome (Fig. 4E). Next, MLDIS was developed by integrating these 36 genes into our integration program.

### Integrated development of a MLDIS

**Fig. 5:**
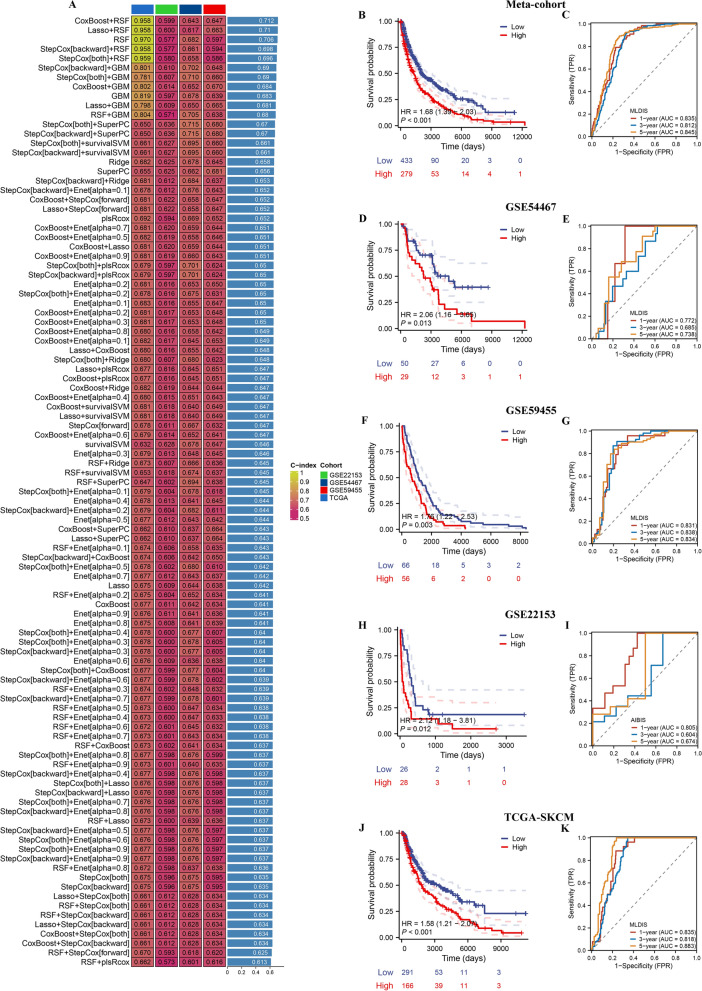
A MLDIS was developed and validated via the machine learning-based integrative procedure. **A** A total of 101 kinds of prediction models via LOOCV framework and further calculated the C-index of each model across all validation datasets. Kaplan–Meier curves of OS according to the MLDIS in meta-cohort (log-rank test: P < 0.001) (**B**), GSE54467 (log-rank test: P = 0.013) (**D**), GSE59455 (log-rank test: P = 0.003) (**F**), GSE22153 (log-rank test: P = 0.012) (**H**), and TCGA-SKCM (log-rank test: P < 0.001) (**J**). Time-dependent ROC analysis for predicting OS at 1, 3, and 5 years in meta-cohort (**C**), GSE54467 (**E**), GSE59455 (**G**), GSE22153 (**I**), and TCGA-SKCM (**K**)

**Fig. 6:**
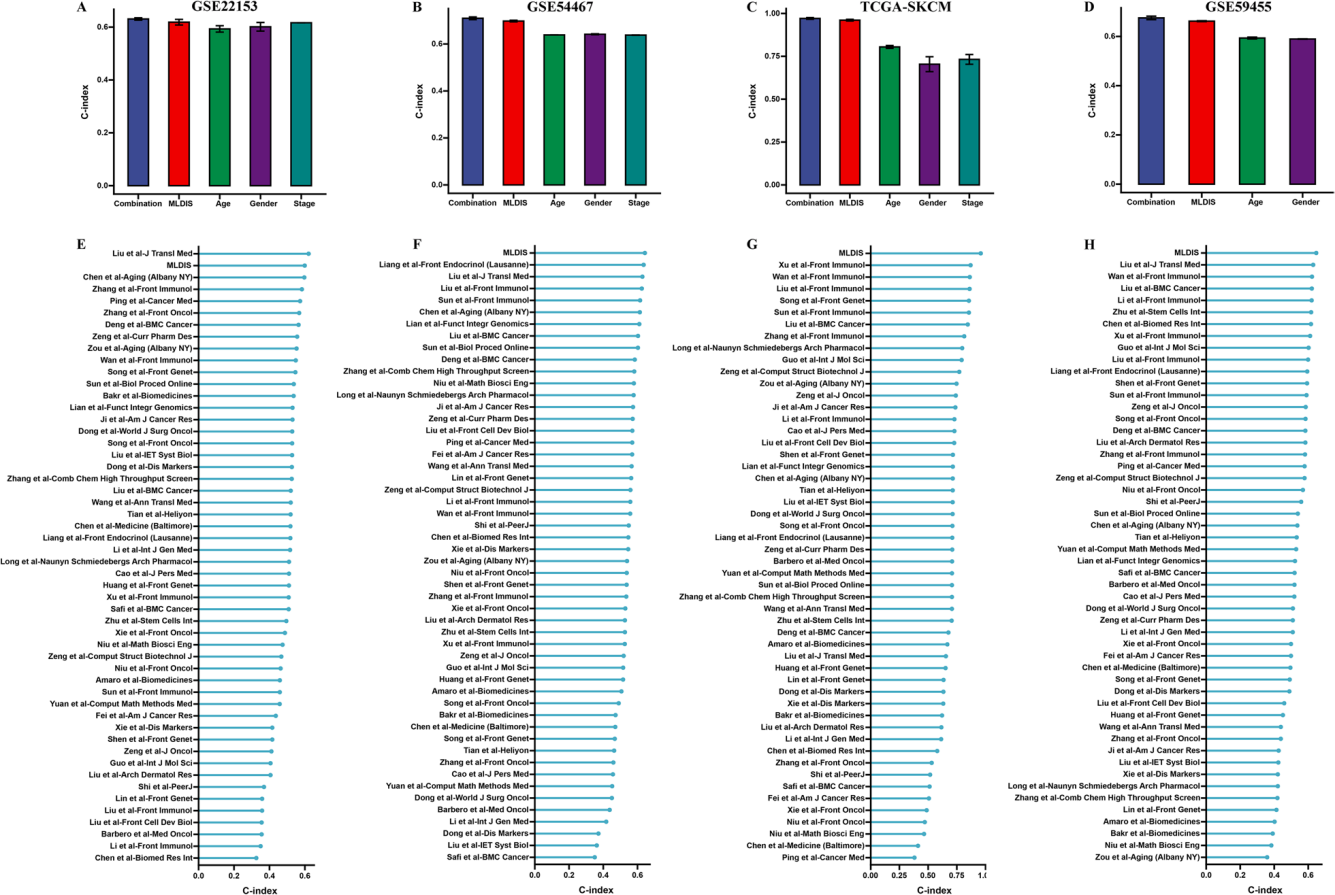
Evaluation of the MLDIS model. The performance of MLDIS was compared with other clinical and molecular variables in predicting prognosis in GSE22153 (**A**), GSE54467 (**B**), TCGA-SKCM (**C**), and GSE59455 (**D**). C-index analysis MLDIS and 51 published signatures in GSE22153 (**E**), GSE54467 (**F**), TCGA-SKCM (**G**), and GSE59455 (**H**)

In the TCGA-SKCM training cohort, we integrated 101 machine learning combinations were used to select the optimal algorithm to construct a MLDIS. According to the average C-index, the combination of CoxBoost + RSF was selected as the final model (C-index = 0.712, Fig. 5A). To explore the relationship between MLDIS and overall survival (OS), we divided melanoma patients into low-MLDIS and high- MLDIS groups and compared the differences in OS between groups. Kaplan–Meier analysis showed that low-MLDIS group had better survival outcomes than high-MLDIS group in meta-cohort (Fig. 5B), GSE54467 (Fig. 5D), GSE59455 (Fig. 5F), GSE22153 (Fig. 5H), and TCGA-SKCM (Fig. 5J). In addition, the predicting OS at 1, 3, and 5 years for meta-cohort was 0.835, 0.812, 0.845 (Fig. 5C), for GSE54467 was 0.772, 0.685, 0.738 (Fig. 5E), for GSE59455 was 0.831, 0.838, 0.834 (Fig. 5G), for GSE22153 was 0.805, 0.604, 0.674 (Fig. 5I), and for TCGA-SKCM was 0.835, 0.818, 0.883 (Fig. 5K). In particular, the MLDIS showed better prognostic accuracy (Fig. 6A–D). These results show that MLDIS has good prediction performance.

### Comparison between the MLDIS and other models in previously 51 published signatures

Genome resequencing is being used to process and detect genetic variants for screening, non-invasive prenatal diagnosis, and cancer diagnosis. Whole exome sequencing is also being used to diagnose patients affected by Mendelian genetic disorders. Gene sequencing technology is expected to become an essential “molecular pathology microscope” for future clinical diagnosis. Individualized whole genome sequencing or targeted region sequencing for disease prevention, diagnosis and treatment is no longer unattainable. Meanwhile, WGCNA, cell type recognition, machine learning algorithms were also developed, it can handle big data such as genomics, transcriptomics, epigenetics, and has already produced a lot of scientific results. Therefore, we summarized 3 years of published prognostic signatures of SKCM to compare the accuracy of MLDIS with these prognostic signatures. We found 51 prognostic signatures for SKCM, and we compared the predictive accuracy of MLDIS with these markers. It is worth noting that MLDIS performs better in each data set than almost all models (Fig. 6E–H).

### Immune landscape of MLDIS

**Fig. 7:**
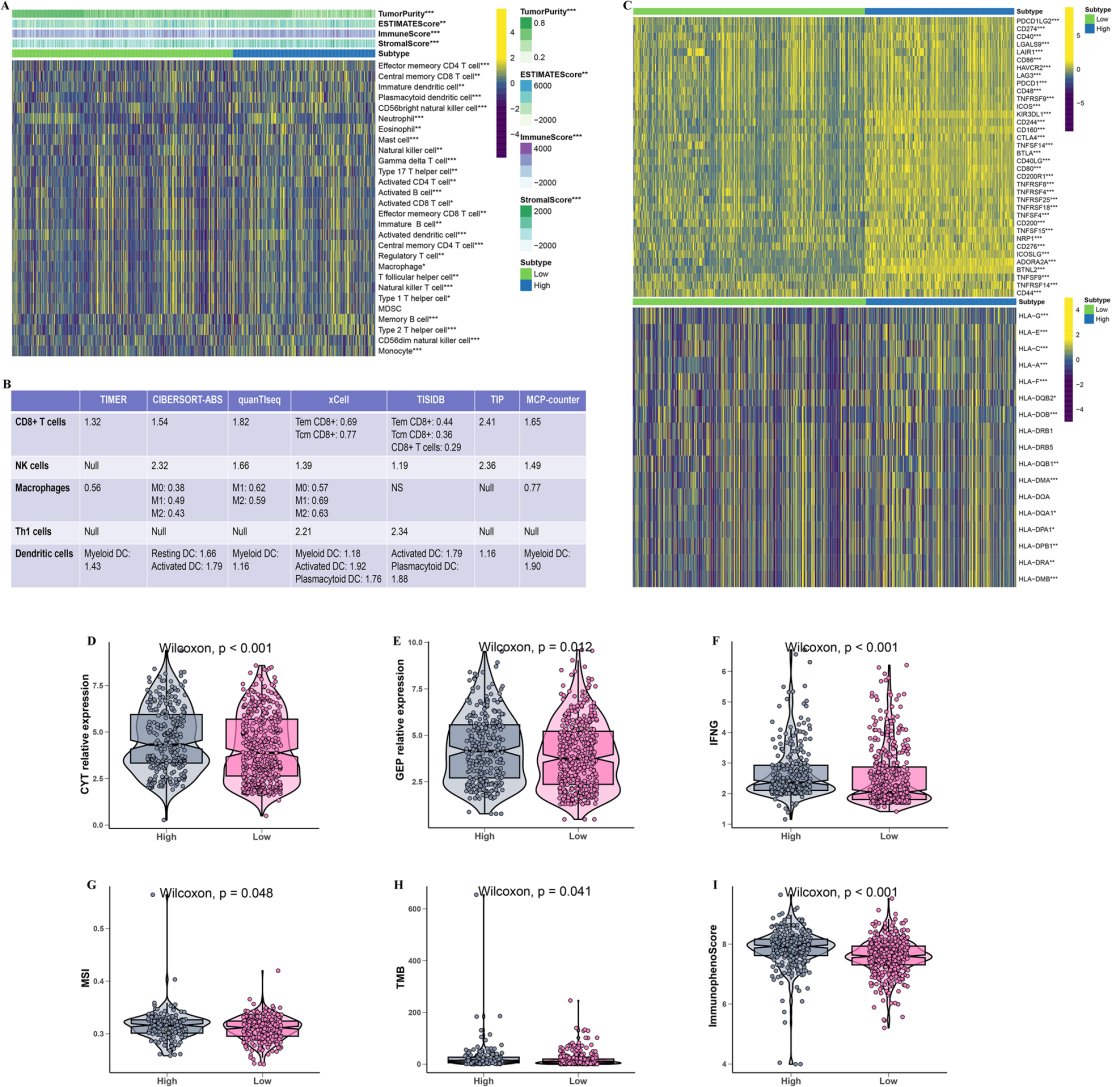
Immune landscape of MLDIS. **A** Heatmap displaying the correlation between the MLDIS and immune infiltrating cells. **B** Correlations between MLDIS and the infiltration levels of five tumor-associated immune cells (CD8 + T cells, NK cells, macrophages, Th1 cells, and dendritic cells). **C** Heatmap displaying the correlation between the MLDIS and immune modulator molecules. **D** Box plot displaying the CYT levels between high and low MLDIS groups. **E** Box plot displaying the GEP levels between high and low MLDIS groups. **F** Box plot displaying the IFN-γ levels between high and low MLDIS groups. **G** Box plot displaying MSI levels between high and low MLDIS groups. **H** Box plot displaying the TMB levels between high and low MLDIS groups. **I** Box plot displaying the IPS levels between high and low MLDIS groups. The asterisks represented the statistical p-value (*P < 0.05; **P < 0.01; ***P < 0.001)

To explore the effect of MLDIS on levels of immune cell infiltration in the SKCM microenvironment, 28 immune cells were evaluated using the ssGSEA method, and then the levels of immune cells in the high-MLDIS and low-MLDIS groups were compared. The results showed that the infiltration level of immune cells in the high-MLDIS group was significantly higher than that in the low-MLDIS group (Fig. 7A). Meanwhile, the stromal score, immune score, and ESTIMATE score were lower in low-MLDIS, but the tumor purity was higher in high-MLDIS group. The infiltrating immune cells in the TME are the key to the anti-tumor effect of the immune system. High levels of infiltration of CD8 + T cells, neutrophil and dendritic cell predict better outcomes and longer survival in immunotherapy patients. Therefore, the correlation between MLDIS and immune cell infiltration was analyzed by using 7 independent algorithms. The results showed that MLDIS was positively correlated with the infiltration levels (Fig. 7B). Thus, it can be speculated that MLDIS may have a positive prognostic impact on patients treated with anti-PD-1 immunotherapy by driving changes in the level of infiltration of immune cells in the TME. Next, to explore the effect of MLDIS on levels of immune checkpoint, and then the levels of immune checkpoint in the high-MLDIS and low-MLDIS groups were compared. The results showed that the expression level of immune checkpoint in the high-MLDIS group was significantly higher than that in the low-MLDIS group (Fig. 7C).

### Immunotherapy response of MLDIS

**Fig. 8:**
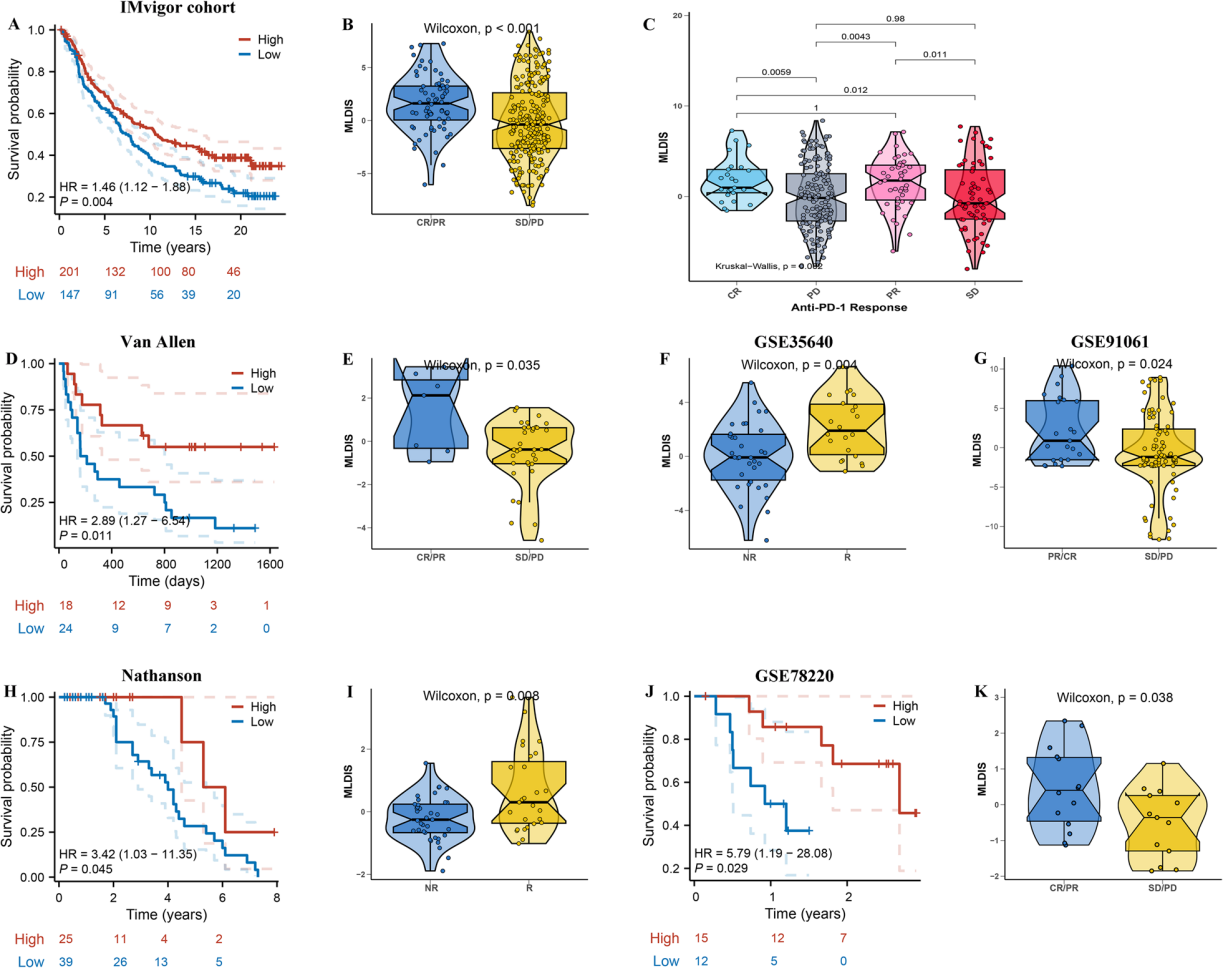
Predictive value of the MLDIS in immunotherapy response. **A** Kaplan–Meier survival curve of OS between patients with a high MLDIS and a low MLDIS in the IMvigor dataset. **B** Box plot displaying the MLDIS in patients with different immunotherapy responses in the IMvigor dataset. **C** Differences in MLDIS among distinct anti-PD-1 clinical response groups. **D** Kaplan–Meier survival curve of OS between patients with a high MLDIS and a low MLDIS in the Van Allen dataset. **E** Box plot displaying the MLDIS in patients with different immunotherapy responses in the Van Allen dataset. **F** Box plot displaying the MLDIS in patients with different immunotherapy responses in the GSE35640 dataset. **G** Box plot displaying the MLDIS in patients with different immunotherapy responses in the GSE91061 dataset. **H** Kaplan–Meier survival curve of OS between patients with a high MLDIS and a low MLDIS in the Nathanson dataset. **I** Box plot displaying the MLDIS in patients with different immunotherapy responses in the Nathanson dataset. **J** Kaplan–Meier survival curve of OS between patients with a high MLDIS and a low MLDIS in the GSE78220 dataset. (K) Box plot displaying the MLDIS in patients with different immunotherapy responses in the GSE78220 dataset

In order to promote the clinical availability of MLDIS, this study investigated the relationship between MLDIS and several immunotherapeutic predictors. Notably, the MSI, TMB, CYT, GEP, immunophenoScore, and IFN-γ levels were all significantly higher in the high MLDIS group (Fig. 7D–I). We further evaluated the potential effect of MLDIS in immunotherapies. The results demonstrated that higher MLDIS subgroup had longer survival time (Fig. 8A, P = 0.004), and SD/PD group had a lower MLDIS than CR/PR group in IMvigor cohort (Fig. 8B and C, P< 0.001). Next, to further validate the robustness of MLDIS in immunotherapy in melanoma, MLDIS model was constructed in 5 melanoma cohort. The OS time curve showed that higher MLDIS subgroup had longer survival time in Van Allen (Fig. 8D, P = 0.011), Nathanson (Fig. 8H, P = 0.045), and GSE78220 (Fig. 8J, P = 0.029). Moreover, the MLDIS was markedly lower in no-response group than that in response group in Van Allen (Fig. 8E, P  = 0.035), GSE35640 (Fig. 8F, P  = 0.004), GSE91061 (Fig. 8G, P  = 0.024), Nathanson (Fig. 8I, P  = 0.008), GSE78220 (Fig. 8K, P  = 0.038). According to these results, the high MLDIS group benefits more from immunotherapy than the low MLDIS group.

### Validation MLDIS in-house cohort

**Fig. 9:**
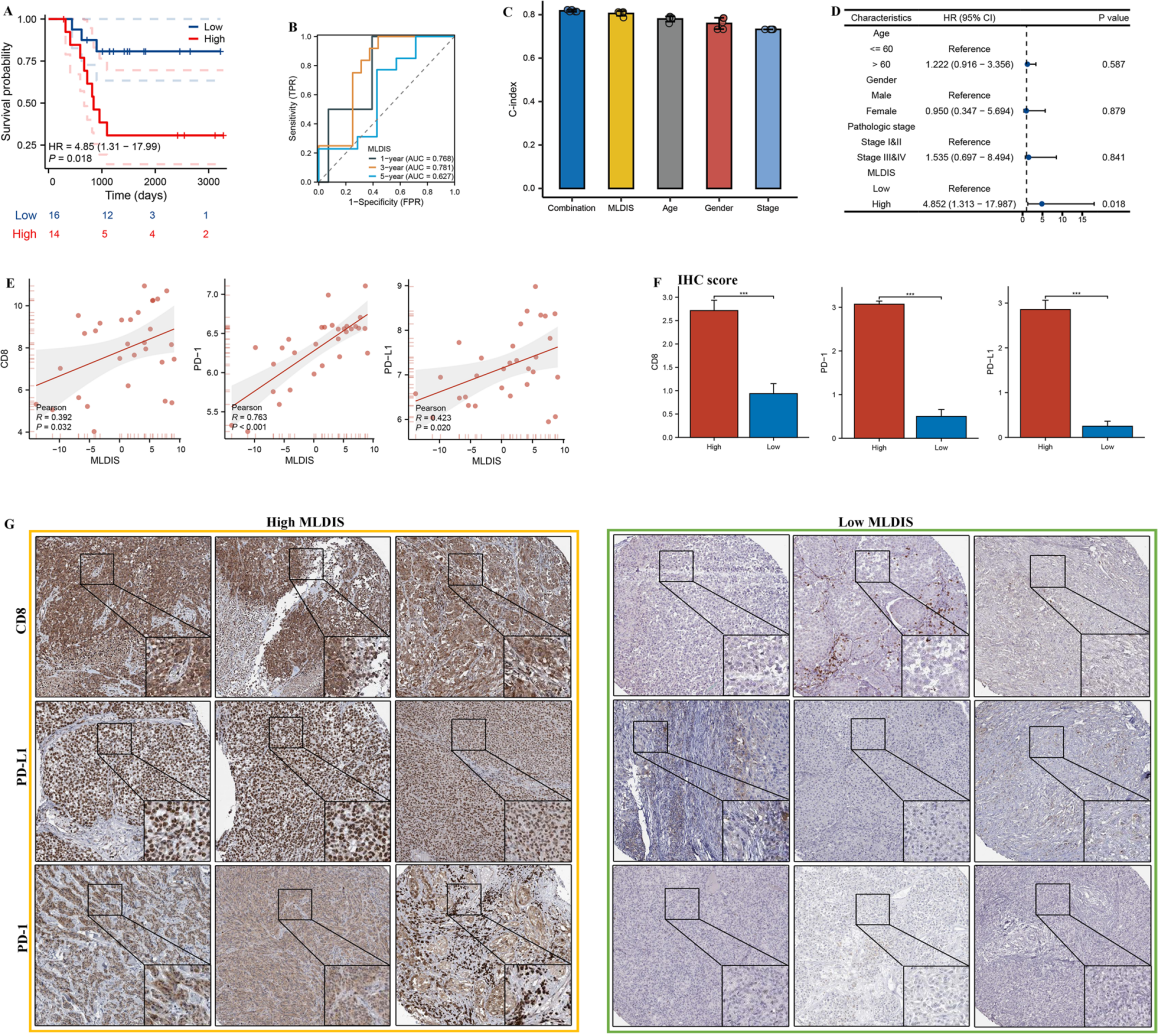
Validation in a clinical in-house cohort. **A** Kaplan–Meier survival curve of OS between patients with a high MLDIS and a low MLDIS in the in-house dataset. **B** Time-dependent ROC analysis for predicting OS at 1, 3, and 5 years in the in-house dataset. **C** The performance of MLDIS was compared with other clinical and molecular variables in predicting prognosis in the in-house dataset. **D** Univariate Cox analysis of OS in the in-house dataset (n = 30). **E** Scatter plot displaying the correlation between the MLDIS and CD8, PD-1, and PD-L1 in the in-house dataset. **F** Box plot displaying the IHC score levels of CD8, PD-1, and PD-L1 based on IHC staining between two MLDIS groups in the MLDIS in-house dataset. **G** Representative IHC staining images of CD8, PD-1, and PD-L1 in two MLDIS groups in the in-house dataset

To further verify the performance of our MLDIS model in a clinically translatable tool, we next evaluated the expression of these RNAs in a clinical cohort of 30 SKCM patients by conducting qRT-PCR assays. Consistently, the OS time in low-MLDIS group was significantly longer than that in high-MLDIS group, and the predicting OS at 1, 3, and 5 years was 0.768, 0.781, 0.627 (Fig. 9A and B). Meanwhile, the MLDIS showed better predictive efficacy in prognosis (Fig. 9C and D). Subsequently, we inspected the correlation between the MLDIS and CD8, PD-1, and PD-L1 in the in-house dataset. The results demonstrated that MLDIS was positive correlation with CD8, PD-1, and PD-L1 (Fig. 9E). Moreover, CD8, PD-1, and PD-L1 were highly expressed in the high-MLDIS group (Fig. 9F and G). Since genes with strong associations may have similar regulatory or biological functions, we hypothesized that this MLDIS is closely related to the efficacy evaluation of anti-PD-1 immunotherapy, it is a potential predictor of the efficacy of anti-PD-1 therapy.

## Discussion

PD-1 immune checkpoint blockade therapy can induce high levels of anti-melanoma response, greatly improving the survival of patients with cancer. However, there are still many patients who cannot benefit from it. PD-L1 expression and TMB level are two predictive markers in anti-PD-1 therapy, which are widely used in clinical tumor immuno-blocking therapy [33, 34]. However, clinical studies and application practice show that these two markers are still controversial in predicting the efficacy of anti-PD-1 therapy. Many patients cannot get the best effect of PD-1 immuno-blocking therapy according to their diagnostic prediction, and even some patients have the opposite response to the therapy. PD-L1 protein expression is not aligned with clinical detection platforms and methodological criteria for TMB levels, which contributes to the inaccuracy of immune blockade therapy predictions based on these two markers [35]. Second, individual treatment-predictive markers often fail to capture the immune status of a patient’s tumor microenvironment comprehensively and accurately. PD-L1 protein expression levels may represent only part of the T cell-related biology. Similarly, TMB only partially represents the ability of neoantigen-reactive T cells to recognize tumor cells. The inherent limitations of a single biomarker make the detection of PD-L1 protein expression with TMB levels insufficient to reveal the complexity of tumor-host immune cell interactions in the TME. It also fails to adequately characterize the patient’s anti-tumor immune status, ultimately leading to bias in the assessment of patient benefit from anti-PD-1 therapy [36]. Thus, to find new markers with better predictive performance and integrate with markers to establish a comprehensive index that can effectively evaluate the anti-tumor immune status of patients.

An innovative computational framework was used in this study in order to identify a robust and stable MLDIS. Firstly, we identified the key immune cell-related gene modules in TCGA-SKCM by using WGCNA, and then after screening and identification, and eventually found 10 hub genes. Secondly, based on 10 hub genes, we identified 2 SKCM subtypes, which have different phenotypes, an immune-desert with higher immune infiltration but poor prognosis, and an immune-excluded subtype with lower immune infiltration but better prognosis. Then, we screen out genes that differed between the 2 SKCM subtypes and had a poor prognosis. Finally, based on the above genes, we build MLDIS by 10 machine learning algorithms.

New computational biology strategies and methods have been used in several studies to comprehensively assess the characteristics of the tumor immune microenvironment, gene function network and clinical phenotype was deeply explored, and a good screening effect of tumor diagnostic and therapeutic predictive markers was obtained. WGCNA-based marker screening tools can effectively identify gene sets with similar expression patterns, highly functionally related genes, and the relationship between gene sets and disease phenotypes. Moreover, WGCNA has a high level and effectiveness in exploring the genetic signature of tumor immune microenvironment that cannot be achieved by other screening tools, due to the effective mapping of the regulatory network of gene sets and the completion of the identification process of key regulatory genes. In this study, we realized a screen for TME markers in SKCM patients and finally identified 132 genes associated with B cells naive. The results of GO and KEGG suggest that these genes play important roles in B cell activation, B cell receptor signaling pathway, and B cell proliferation. This fully demonstrates and validates the reliability and validity of our transcriptome-based computational biology combined with WGCNA to form a robust, rigorous screening process approach.

Although WGCNA provided a robust strategy for marker screening in the initial process, and as a result, 10 potential efficacy prognostic markers were obtained, we should be conscious of the fact that the clinical efficacy of anti-PD-1 immunotherapy is influenced by multiple factors, and not reliance on a single predictive. The results of MLDIS-immune cell infiltration-based analysis provide evidence to support the prognostic predictive value of MLDIS at the level of the tumor immune microenvironment. This study also found a significant correlation between MLDIS and immune scores, suggesting that immune function exerts a significant influence on the risk of melanoma death. M1 macrophages in melanoma are associated with improved prognosis of melanoma [37], and NK cells induce M1 polarization and inhibit tumor growth [38]. Similarly, the present study observed higher immune scores and higher abundance of M1 macrophages, CD4 + T cells, CD8 + T cells and NK cells in the high-MLDIS group, further suggesting the influence of these tumor-infiltrating immune cells on melanoma development. High levels of infiltration of CD8 + T cells, CD4 + T cells, neutrophils, B cells, M1-polarized macrophages and dendritic cells determine the pre-existing anti-tumor immune activity of patients and are associated with better prognosis and longer survival in patients receiving immunotherapy [39–41]. MLDIS correlated significantly with the efficacy of anti-PD-1 therapy, and their high expression levels predicted good prognostic benefit. In addition, MLDIS positively correlates with PD-L1 expression levels, and they may positively influence anti-PD-1 immunotherapy by regulating the infiltration status of immune cells in the tumor microenvironment through similar regulatory or functional biological effects [42].

With the massive generation of sequencing data such as human genome resequencing and sequence alignment, it is necessary to take full advantage to develop and study important genetic loci in tumorigenesis and development. This bioinformatics-based approach to analyze tumorigenesis has the advantage of being more efficient, flexible, and targeted than traditional biological research methods. As sequencing technology continues to evolve, we believe our model has great potential for clinical practice. Although an efficacy assessment model for MLDIS markers was constructed, there are still problems and limitations associated with it. First, although this study used computational biology to validate the potential efficacy predictive markers of MLDIS from multiple perspectives, further biological validation, especially in large-scale clinical trials, is lacking, which is important for the final validation of biomarker reliability. Secondly, the limited number of training samples currently used for deep learning in this study may result in less-than-optimal performance of the resulting evaluation model in predicting the efficacy of anti-PD-1 therapy in other patient cohorts. Therefore, in the future, collecting more SKCM-related datasets and continuously optimizing the prediction models are the directions for further research towards personalized immunotherapy and clinical applications.

## Conclusion

In conclusion, we conducted a MLDIS model by using 10 machine learning algorithms (101 combinations). In addition to the expression of immune checkpoint genes, immune cell infiltrations in high and low MLDIS groups were also explored. Meanwhile, the MLDIS model can facilitate the prediction and the selection of SKCM individual and personalized immunotherapeutic.

### Supplementary Information

**Additional file 1: Table S1.** Clinical characteristics of the 30 SKCM patients used in this study.

**Additional file 2: Table S2.** The sequence of primers was used in this study.

**Additional file 3: Table S3.** List of 132 module genes used in this study.

**Additional file 4: Table S4.** Differentially expressed genes between 2 clusters in the meta-cohort.

**Additional file 5: Figure S1.** WGCNA analysis. (A and B) The heatmap revealed the eigengene adjacency of modules.

**Additional file 6: Figure S2.** Differentially expressed module genes between tumor and normal tissue in TCGA-SKCM dataset.

## Data Availability

The datasets generated for this study can be found in the GEO database (GSE22153, GSE54467, GSE59455, GSE35640, GSE91061, GSE78220, Van Allen, and Nathanson; https://www.ncbi.nlm.nih.gov/geo/), and UCSC Xena website (https://gdc.xenahubs.net).
