# Supplementary figures and images for: Machine learning-derived identification of tumor-infiltrating immune cell-related signature for improving prognosis and immunotherapy responses in patients with skin cutaneous melanoma
Source: Cancer Cell Int. 2023 Sep 26;23:214. doi: 10.1186/s12935-023-03048-9 (PMC10521465; doi:10.1186/s12935-023-03048-9)

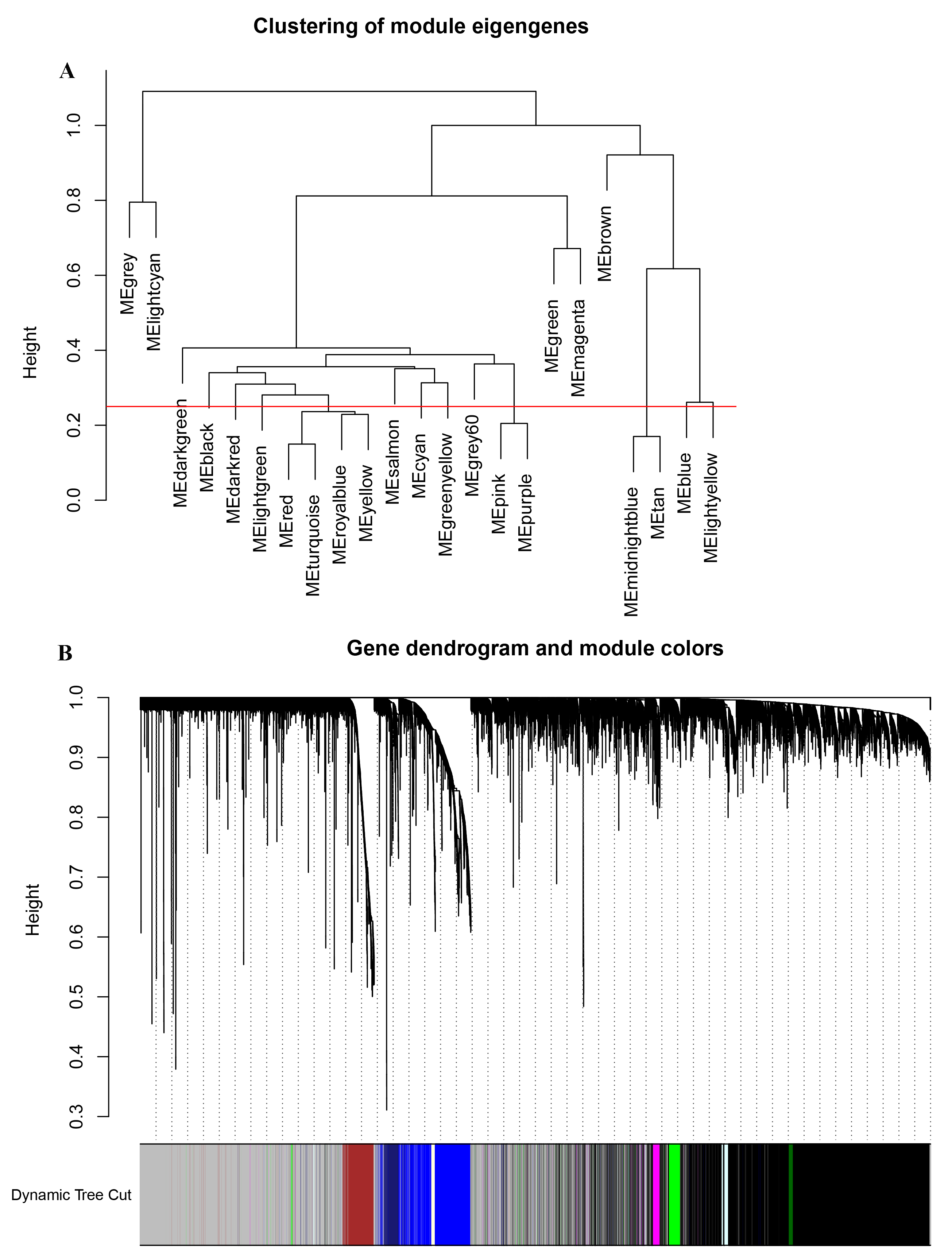

Supplement: Supplementary file 5 — Additional file 5: Figure S1. WGCNA analysis. (A and B) The heatmap revealed the eigengene adjacency of modules. [file 12935_2023_3048_MOESM5_ESM.tif]

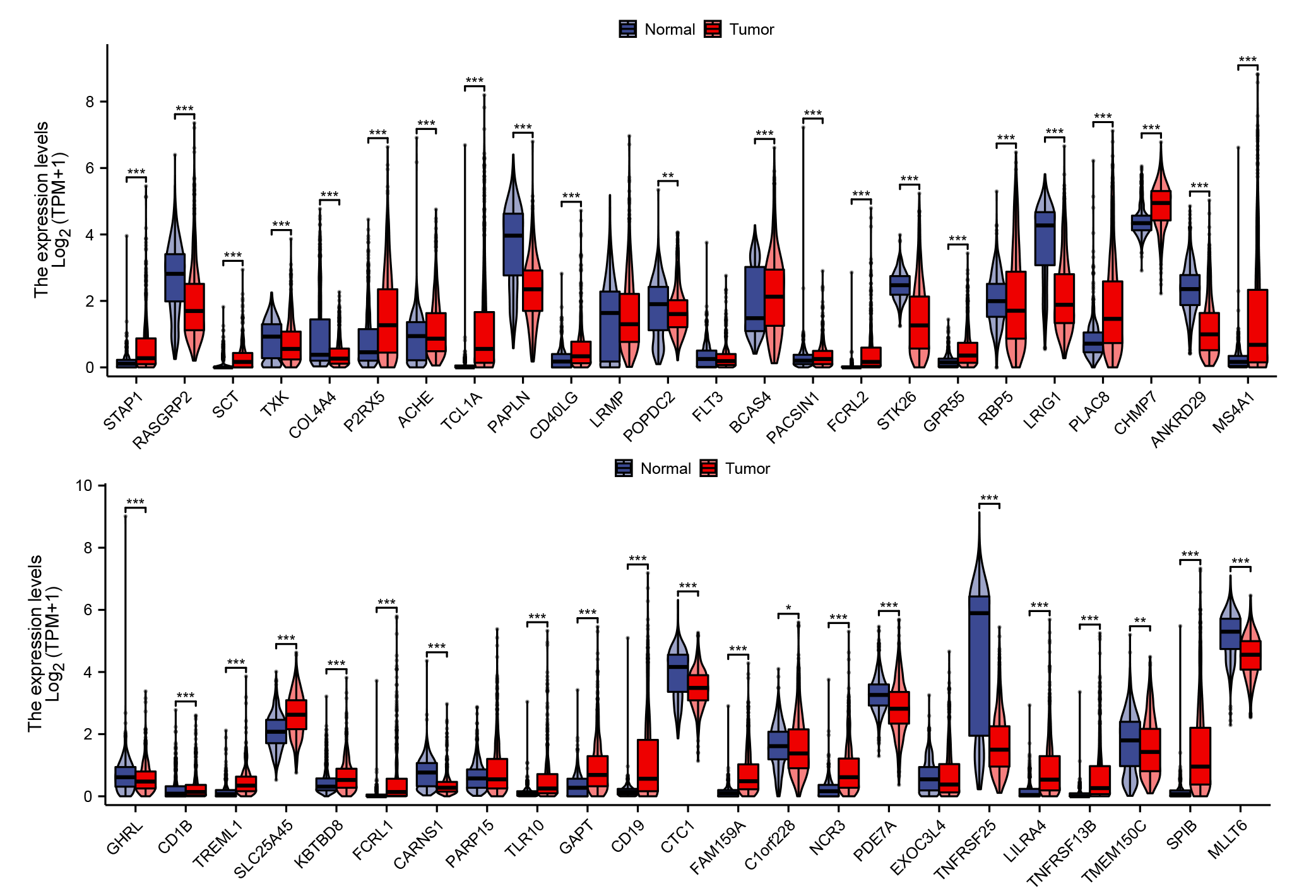

Supplement: Supplementary file 6 — Additional file 6: Figure S2. Differentially expressed module genes between tumor and normal tissue in TCGA-SKCM dataset. [file 12935_2023_3048_MOESM6_ESM.tif]
